# Surgical resection does not avoid the risk of diverticulitis recurrence—a systematic review of risk factors

**DOI:** 10.1007/s00384-020-03762-0

**Published:** 2020-09-28

**Authors:** Gregoire Longchamp, Ziad Abbassi, Jeremy Meyer, Christian Toso, Nicolas C. Buchs, Frederic Ris

**Affiliations:** Division of Digestive Surgery, University Hospitals of Geneva, Rue Gabrielle-PerreT-Gentil 4, 1211 Geneva, Switzerland

**Keywords:** Diverticulitis, Sigmoidectomy, Postoperative recurrence, Risk factors

## Abstract

**Purpose:**

Fifteen percent of patients undergoing elective sigmoidectomy will present a diverticulitis recurrence, which is associated with significant costs and morbidity. We aimed to systematically review the risk factors associated with recurrence after elective sigmoidectomy.

**Methods:**

PubMed/MEDLINE, Embase, Cochrane, and Web of Science were searched for studies published until May 1, 2020. Original studies were included if (i) they included patients undergoing sigmoidectomy for diverticular disease, (ii) they reported postoperative recurrent diverticulitis, and (iii) they analyzed ≥ 1 variable associated with recurrence. The primary outcome was the risk factors for recurrence of diverticulitis after sigmoidectomy.

**Results:**

From the 1463 studies initially screened, six studies were included. From the 1062 patients included, 62 patients recurred (5.8%), and six variables were associated with recurrence. Two were preoperative: age (HR = 0.96, *p* = 0.02) and irritable bowel syndrome (33.3% with recurrence *versus* 12.1% without recurrence, *p* = 0.02). Two were operative factors: uncomplicated recurrent diverticulitis as indication for surgery (73.3% with recurrence *versus* 49.9% without recurrence, *p* = 0.049) and anastomotic level (colorectal: HR = 11.4, *p* = 0.02, or colosigmoid: OR = 4, *p* = 0.033). Two were postoperative variables: the absence of active diverticulitis on pathology (39.6% with recurrence *versus* 26.6% without recurrence) and persistence of postoperative pain (HR = 4.8, *p* < 0.01).

**Conclusion:**

Identification of preoperative variables that predict the occurrence of diverticulitis recurrence should help surgical decision-making for elective sigmoidectomy, while peri- and postoperative factors should be taken into account for optimal patient follow-up.

**Electronic supplementary material:**

The online version of this article (10.1007/s00384-020-03762-0) contains supplementary material, which is available to authorized users.

## Introduction

Diverticulosis is defined by the presence of colonic diverticula which are protrusions of the mucosa and submucosa through the colonic wall. More than 90% of colonic diverticula are found in the left colon and sigmoid [1]. Based on an American population aged between 30 and 80 years undergoing outpatient colonoscopies, diverticulosis was present in 42% of patients [2]. This prevalence was increased in elderly, white population, overweight, smokers, and patients with decreased bowel movements [2]. Patients may remain asymptomatic, whereas others will develop diverticular disease, defined as symptomatic diverticulosis. Therefore 10–25% of patients with diverticulosis will manifest diverticular inflammation, and 12% of patients with diverticulitis will developed a complication such as abscess, perforation, fistula, stricture or obstruction [3].

The Hinchey classification modified by Wasvary et al. [4] is often used to classify severity of episode of diverticulitis. That classification includes four stages: stage Ia corresponds to a confined inflammation or phlegmon; stage Ib is characterized by a pericolic or mesenteric abscess; stage II is characterized by a distant abscess in the abdomen, pelvis, or retroperitoneum; and perforation leading to purulent or fecal peritonitis correspond to stages III or IV, respectively.

The European Association for Endoscopic Surgery (EAES) and other interventional techniques and Society of American Gastrointestinal and Endoscopic Surgeons (SAGES) published guidelines in 2019 [5] recommending emergent sigmoid resection for Hinchey III and IV diverticulitis or after failure of conservative therapies for earlier stages. Elective sigmoidectomy was recommended in the case of decreased quality of life caused by diverticular disease. Moreover, chronic symptoms or smoldering disease, severity of prior episodes, comorbidities, and patient preferences should be taken into consideration [6].

Nevertheless, sigmoidectomy, although removing the segment of the colon the most affected by diverticula, as well as the recto-sigmoid junction, does not remove diverticula from the remaining colon. After a mean follow-up of 10 years, a recurrence rate of 15% after elective surgery for diverticulitis was reported [7]. Mechanism for these recurrences is not clear. However, several risk factors were identified. Prediction of these recurrences is important to prevent associated costs and morbidity [8]. Therefore, we aimed to systematically review the risk factors associated with recurrence of diverticulitis after elective sigmoidectomy.

## Materials and methods

This systematic review was performed in accordance with the recommendations of the Preferred Reporting Items for Systematic Review and Meta-analyses (PRISMA) statement [9] (Supplementary Table 1).

### Data source and search strategy

Two reviewers (GL, ZA) independently searched PubMed/MEDLINE, Embase, Cochrane, and Web of Science for studies published until May 1, 2020, without limitation based on the publication year. The following search terms were used: “diverticulitis” OR “diverticulum” AND “inflammation”, AND “surgery” OR “colectomy”, AND “recurrence” in MeSH terms; and “diverticula” OR “diverticulosis”, AND “resection” OR “sigmoidectomy” OR “Hartmann*”, AND “recurrent” OR “failure” in non-MeSH terms. Additionally, a manual cross-reference search of bibliographies of relevant articles was performed to identify additional studies.

### Study selection

Original studies written in English were eligible for inclusion if they fulfilled all the following criteria: (i) they included patients undergoing elective sigmoidectomy for diverticular disease, (ii) they reported postoperative recurrent diverticulitis, and (iii) they reported ≥ 1 variable associated with recurrence. Studies were excluded if postoperative recurrence was not confirmed by imaging or if the definition of recurrence was not specified. Studies including surgical procedure without resection (i.e., peritoneal lavage, surgical drainage) were excluded. Other exclusion criteria were case reports, conference abstracts, editorials, and protocols. There was no restriction based on the design or sample size of the study.

### Data extraction

Two authors (GL, ZA) independently extracted the data, including general and methodological information of the study and baseline characteristics of the study population: sample size, age, gender, classification of diverticulitis, the number of previous episodes of diverticulitis, and indication for surgery. Intraoperative data were also extracted, including elective/emergency intervention, type of resection (sigmoidectomy/left-sided hemicolectomy/anterior resection), splenic flexure mobilization, laparoscopic/open resection, conversion, creation of ostomy, and type of anastomosis (stapled/handsewn, colorectal/colosigmoid). Postoperative extracted data were follow-up duration, pathology report (specimen length, inflammation state [active, chronic, none]), persistent complaints, complications, recurrence, and treatment for recurrence. Variables associated with recurrence on quantitative analysis and variables significant on uni- or multivariate regression analysis were also extracted.

### Outcome measures

The primary outcome of the systematic review was to identify risk factors for postoperative recurrence of diverticulitis. The secondary outcomes were the incidence of postoperative recurrence of diverticulitis, treatment for postoperative recurrence of diverticulitis (medical versus surgical), postoperative complications, and mortality.

Recurrence was defined as left lower quadrant pain, inflammation (fever, elevated white blood cell, or C-reactive protein), and imaging consistent with the diagnosis of diverticulitis. Complication was defined as any deviation from the normal postoperative course and did not include recurrence.

## Results

### Studies selection and characteristics

**Fig. 1:**
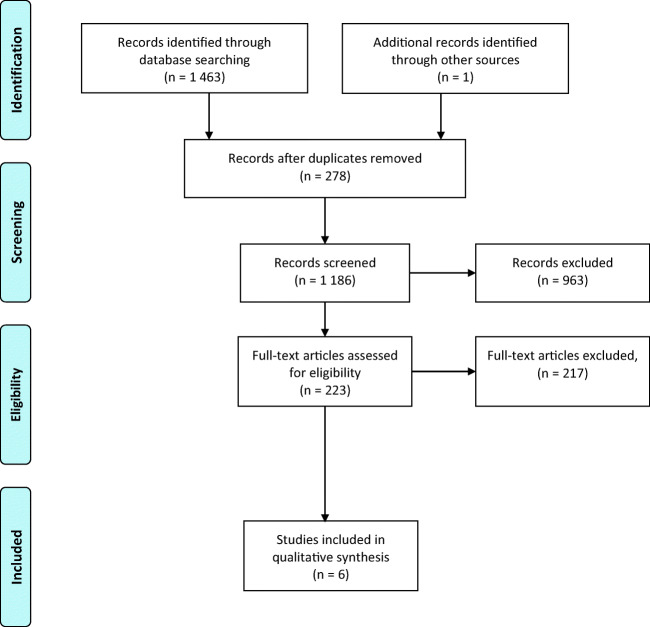
Preferred Reporting Items for Systematic Review and Meta-analyses (PRISMA) flowchart showing selection of publications for review

The initial search identified 1463 studies (Fig. 1). After duplicates removal, 1186 records were screened. Based on the title and abstract, 963 studies were removed. From the 223 full text articles assessed for eligibility, 208 were excluded because they did not fulfill all inclusion criteria. Furthermore, nine other studies were removed: one study [10] contained duplicated data from another included study; two studies [11, 12] included peritoneal lavage, surgical drainage, or diverticulectomy in the resection group; in five studies [13–17] recurrences were not defined or confirmed by imaging; and one study [18] contained insufficient data. One study [19] was identified by cross-referencing. Finally, six articles [19–24] were included in the present review.

From the included studies, five studies were monocentric [19–23] and retrospective observational cohort [19–22, 24] (Table 1). Overall, 1062 patients were included (518 males and 544 females) with a mean age ranging from 56 to 63 years. Data regarding the mean follow-up duration was reported by 4 studies [19–21, 24], which ranged from 55 to 86 months. Before the surgical procedure, 765 (72%) patients experienced recurrent attacks of diverticulitis, and 128 (12%) presented their first episode (data unavailable for 169 (16%) of cases). Only two studies [22, 23] reported the severity of the diverticulitis episode, classified either with the Hinchey classification [25] or with the Hansen and Stock classification [26].

**Table 1:** Characteristics of included studies and baseline characteristics of participants

Study	Methodological characteristics				Population characteristics									
	Journal of publication	Study design	Monocentric or multicentric	Study period	Sample size	Age (mean ± SD or range)	Gender (male/female)	Follow-up (mean ± SD or range)	Diverticulitis severity	Previous episode of diverticulitis				
										0	1	2	3	≥ 4
Bergamaschi et al. 1998, France	Surgical endoscopy	ORC	Monocentric	1990–1994	75	60.9	48/27	55 months	-ª	-				
Thaler et al. 2003, USA	Disease of the colon & rectum	ORC	Multicentric	1992–2000	236	60.4 ± 10	131/105	67 ± 30 months	-°	1*	62*	113*	10*	0
Regenet et al. 2005, France	Hepatogastroenterology	OPC + ORC	Monocentric	1996–2001	94	56.4	47/47	NA	Hinchey classification: 72 patients stage 0 22 patients stage 1	-				
Andeweg et al. 2008, Netherlands	World journal of surgery	ORC	Monocentric	1985–2003	183	63, 26–93	84/99	86, 0–216 months	-	63	16	88	11	5
Holmer et al. 2011, Germany	Langenbeck’s archives of surgery	OPC	Monocentric	2004–2008	113	62.4, 38–92^∏^	46/67	NA	Hansen and Stock classification: 12 patients stage I 59 patients stage 2a 42 patients stage 2b	45	68: ≥ 1 episode			
Choi et al. 2019, USA	Journal of gastrointestinal surgery	ORC	Monocentric	2002–2016	361	56 ± 10.7	162/199	86, 6–190 months	-	19	214: 1–3 episodes			128

*SD* standard deviation, *ORC* observational retrospective cohort, *-* not available, *OPC* observational prospective cohort

*Based on the previous admission, data not available for all patients but 235 cases with more than one episode of diverticulitis

^∏^expressed as median, with range

ªComplicated diverticulitis were excluded

°Perforated diverticulitis were excluded

### Perioperative and postoperative outcomes

Only one study [20] included emergency surgical resections, representing 73 (7%) of patients included in the review (Table 2). The remaining patients represented 953 (93%) cases, which underwent elective intervention for uncomplicated diverticulitis [19–21, 24] or complicated diverticulitis [20–22]. Other reported indications for surgery were symptomatic diverticulosis [23], persistent abdominal pain/smoldering disease [21, 22], recurrent bleeding [22], failure of conservative treatment for diverticulitis [22], and first attack of diverticulitis in immunosuppressed patients [22]. Sigmoidectomy was the procedure of choice in 928 (90%) patients, while anterior resection or left-sided hemicolectomy were not routinely performed (11 patients and 38 patients, respectively; type of intervention performed not reported for 85 patients). Details of the operative techniques are detailed in Table 2.

**Table 2:** Operative characteristics

Study	Indication for surgery	Elective/emergency	Type of operation		Laparoscopic/open// conversion	Splenic flexure mobilization	Ostomy creation	Anastomosis	
			Sigmoidectomy	Other				Stapler/Handswen	Colorectal/Colosigmoid
Bergamaschi et al.	Uncomplicated diverticulitis	75/0	-		40/35 // 1	46	0	51/24	50/25
Thaler et al.	Uncomplicated diverticulitis	236/0	236	0	96/140 // 18	109	0	171 / 65	143 / 93
Regenet et al.	Symptomatic diverticulosis	94/0	94	0	72/22 // 0	94	0	94/0	94/0
Andeweg et al.	Uncomplicated and complicated diverticulitis	110/73	150	5 LH 18 AR*	-	-	68	-	21/90^¥^
Holmer et al.	Persistent abdominal pain Covered perforation ± abscess Recurrent bleeding Failure of conservative treatment First attack in immunosuppressed	113/0	113	0	-	113	0	113/0	-
Choi et al.	Uncomplicated recurrent diverticulitis Localized perforation ± abscess Fistula Stricture Smoldering disease	361/0	335	6 LH 20 AR	359 / 2 // 41	0	5	361/0	361/0

*-* not available, *LH* left-sided hemicolectomy, *AR* anterior resection

*10 classified as miscellaneous

^¥^4 anastomosis classified as other

Pathology showed a mean specimen length from 14 to 26 cm [19–24] and the presence of diverticulitis in 536 cases, but no inflammation in 177 cases [19–21, 23] (Table 3). Moreover, inflammation at the proximal margin was reported by two studies in 13 [19] and 30 [24] patients. Data on the presence of postoperative persistent complaints was reported by one study [20] in 36 patients. Five studies [19–22, 24] reported postoperative complications, corresponding to a total of 136 events and an incidence of 14.0% (136/968); and four studies [19, 20, 22, 24] reported postoperative death, corresponding to 8 patients and a mortality of 1.3% (8/607).

**Table 3:** Postoperative outcomes

Study	Pathology		Complications (timepoint)	Persistent complains	Mortality (timepoint)
	Specimen length (mean ± SD or range)	Pathologist assessment			
Bergamaschi et al.	14.3 cm	75 diverticulitis^∏^	8	-	0
Thaler et al.	17.9 ± 5.9 cm	-^∏^	54 (30 days)	-	1 (30 days)
Regenet et al.	26.3 cm	94 diverticulitis	-	-	-
Andeweg et al.	17.3, 7-35 cm	166 acute diverticulitis 17 no inflammation	9*	36	7
Holmer et al.	-	-	16	-	0
Choi et al.	17.7 cm	141 acute diverticulitis 60 chronic diverticulitis 158 diverticular disease 2 no disease	49 (perioperative)	-	-

*SD* standard deviation, *cm* centimeter, - not available

^∏^inflammation at the proximal margin was reported in 13 cases by Bergamaschi et al. and in 30 cases by Thaler et al.

*only severe complications were reported

### Postoperative recurrence of diverticulitis

A total of 62 diverticulitis recurred after surgical resection for diverticular disease, representing 5.8% of the population included in the review. Mean time to recurrence ranged between 38 [20] and 78 months [24], and the cumulative time-related incidence at 15 years ranged between 6.3 [21] and16% [20] (Table 4). The treatment for postoperative recurrence was conservative in 43 patients, but 14 patients required another surgical intervention (treatment not reported by one study [23]).

**Table 4:** Reported recurrences of included studies

Study	Number of recurrence (%)	Time until recurrence (mean ± SD or range)	Cumulative time-related incidence of recurrence	Treatment for recurrent diverticulitis	
				Conservative	Surgical
Bergamaschi et al.	4 (5.3%)	-	-	4	0
Thaler et al.	12 (5.1%)	78 ± 25 months	-	11	1
Regenet et al.	5 (5.3%)	-	-	-	
Andeweg et al.	16 (8.7%)	38, 6–144 months	1 year: 3% (SE 1.3) 5 years: 8.2% (SE 2.3) 10 years: 12% (SE 3.0) 15 years: 16% (SE 3.7)	8	8^∏^
Holmer et al.	4 (3.5%)	-	-	4	0
Choi et al.	21 (5.8%)	55, 6–109 months	1 year: 0.3% 5 years: 3.0% 10 years: 6.3% 15 years: 6.3%	16	5*
Overall	62 (5.8%)	-	-	43	14

*SD* standard deviation, - not available, *SE* standard error

^∏^3 left hemicolectomy, 3 partial resection of transverse colon, 2 subtotal colectomy

*5 left colectomies

#### Preoperative variables associated with postoperative recurrence of diverticulitis

Eight preoperative variables were considered for their association with recurrence of postoperative diverticulitis (Table 5). Age was not associated with postoperative recurrence in the retrospective study by Choi et al. [21] (*p* = 0.12). However, Andeweg et al. [20] reported a lower age to be associated with recurrence (mean 54, range 33–75, *versus* without recurrence: mean 64, range 27–93, *p* < 0.02). On regression analysis (Cox-model), younger age was still an independent predictor of recurrence (univariate: hazard ratio (HR) = 0.96, 95% CI 0.93–0.99, *p* = 0.02; multivariate: stated as significant but no value reported). Irritable bowel syndrome was the other preoperative variable associated with recurrence on univariate analysis (33.3% with recurrence *versus* 12.1% without recurrence, *p* = 0.02) [21]. Nevertheless, the latter was not significant on regression analysis (*p* = 0.053). Six preoperative variables showed no significant association with recurrence: the number of preoperative episodes of diverticulitis (reported by four studies [20–22, 24]), gender (reported by three studies [20, 21, 24]), American society of anesthesiologists (ASA) class and previous abdominal surgery (both reported by one study [24]), comorbidity, and previous treatment modality (both reported by one study [21]).

**Table 5:** Preoperative variables associated with postoperative recurrence of diverticulitis

Study	Univariate analysis	Univariate regression analysis	Multivariate regression analysis
Number of preoperative episodes of diverticulitis			
Thaler et al.	-	NS	-
Andeweg et al.	NS	NS	-
Choi et al.	NS	-	-
Holmer et al.	NS	-	-
Gender			
Thaler et al.	-	NS	-
Andeweg et al.	NS	-	-
Choi et al.	NS	-	-
Age: mean, range with recurrence *versus* without recurrence			
Andeweg et al.	54, 33–75 *versus* 64, 27–93, *p* < 0.02	HR 0.96, 95% CI 0.93–0.99, *p* = 0.02	Significant (value NA)
Choi et al.	NS	-	-
ASA class			
Thaler et al.	-	NS	-
Comorbidity			
Choi et al.	NS	-	-
Irritable bowel syndrome: *n* (%) with recurrence *versus* without recurrence			
Choi et al.	5 (33.3%) versus 42 (12.1%), *p* = 0.02	NS	-
Previous treatment modality: antibiotic IV *versus* antibiotic PO *versus* drainage			
Choi et al.	NS	-	-
Previous abdominal surgery			
Thaler et al.	-	NS	-

*NS* not significant, *ASA* American Society of Anesthesiologists, *IV* intravenous, *PO* per os, - not available

#### Operative variables associated with postoperative recurrence of diverticulitis

From the eight operative variables, only two [20, 21, 24] were associated with recurrence (Table 6). The first variable was uncomplicated recurrent diverticulitis as indication for surgery (73.3% with recurrence *versus* 49.9% without recurrence, *p* = 0.049), but the association was not significant on univariate regression analysis [21]. Anastomotic level was considered by three studies [20, 23, 24], but significant in two studies [20, 24] on univariate regression analysis. Andeweg et al. [20] reported increased recurrences associated with colorectal anastomosis compared with colostomy (univariate regression analysis: HR = 11.4, 95% CI 1.2-109.5, *p* = 0.02; multivariate regression analysis: stated as significant but no value reported). In the other hand, Thaler et al. [24] reported colosigmoid anastomosis as a risk factor for postoperative recurrent diverticulitis (univariate regression analysis: odds ratio (OR) = 4, 95%CI 1.1-15.0, *p* = 0.033; no multivariate regression analysis). Six other factors were not associated with postoperative recurrence of diverticulitis: emergency/elective surgery [20], laparoscopic/open approach [19, 23, 24], length of resected bowel [20, 21, 23], type of resection [20, 21], splenic flexure mobilization [24], stapled/handsewn anastomosis [24].

**Table 6:** Operative variables associated with recurrence associated with postoperative recurrence of diverticulitis

Study	Univariate analysis	Univariate regression analysis	Multivariate regression analysis
Unomplicated recurrent diverticulitis as indication for surgery: n (%) with recurrence *versus* without recurrence			
Choi et al.	11 (73.3) *versus* 171 (49.9%), *p* = 0.049	NS	-
Emergency *versus* elective surgery			
Andeweg et al.	NS	-	-
Laparoscopic *versus* open			
Thaler et al.	-	NS	-
Regenet et al.	NS	-	-
Bergamaschi et al.	NS	-	-
Anastomotic level: n (%) of recurrence			
Andeweg et al.	Colorectal: 3 (14.3%), colosigmoid: 12 (13.3%) *versus* colostomy 1 (1.5%), *p* = 0.04	Colorectal: HR 11.4, 95% CI 1.2-109.5, *p* = 0.02, colosigmoid: NS	Colorectal: Significant (value NA)
Thaler et al.	-	Colosigmoid: 7 (12.5%) *versus* colorectal: 4 (2.8%); OR = 4, 95% CI 1.1–15.0, *p* = 0.033	-
Regenet et al.	NS	-	-
Length of resected bowel			
Choi et al.	NS	-	-
Andeweg et al.	NS	-	-
Regenet et al.	NS	-	-
Type of resection: sigmoidectomy versus anterior resection *versus* left-sided hemicolectomy			
Choi et al.	NS	-	-
Andeweg et al.	NS	-	-
Splenic flexure mobilization			
Thaler et al.	NS	-	-
Anastomotic technique: stapled *versus* handsewn			
Thaler et al.	NS	-	-

*NS* not significant

- not available

#### Postoperative variables associated with postoperative recurrence of diverticulitis

Four postoperative variables were included in the analysis for their association with diverticulitis (Table 7). The absence of active diverticulitis on pathology was significant on univariate analysis only in the study by Choi et al. [21] (39.6% with recurrence *versus* 26.6% without recurrence, *p* = 0.01). However, two studies [20, 24] reported no association between the pathology and the recurrence of postoperative diverticulitis. Persistence of postoperative pain was associated with recurrence on univariate analysis but also on uni- and multivariate regression analysis (22% with recurrence *versus* 5.4% without recurrence, *p* < 0.01; HR = 4.8, 95% CI 1.8–12.5, *p* < 0.01; stated as significant but no value reported; respectively) [20]. Two postoperative factors were not associated with recurrence, as reported by one study [24]: postoperative complications and reoperation.

**Table 7:** Postoperative variables associated with recurrence associated with postoperative recurrence of diverticulitis

Study	Univariate analysis	Univariate regression analysis	Multivariate regression analysis
Acute diverticulitis on pathology: *n* (%) with recurrence *versus* without recurrence			
Choi et al.	4 (26.6%) *versus* 137 (39.6%), *p* = 0.01	NS	-
Andeweg et al.	NS	-	-
Thaler et al.	-	NS^∏^	-
Persistent postoperative pain: n (%) of recurrences in patients with persistent pain *versus* n (%) of recurrences without persistent pain			
Andeweg et al.	8 (22.2%) *versus* 8 (5.4%), *p* < 0.01	HR 4.8, 95% CI 1.8–12.5, *p* < 0.01	Significant (value NA)
Postoperative complication			
Thaler et al.	-	NS	-
Reoperation			
Thaler et al.	-	NS	-

*NS* not significant

- not available

^∏^described as inflammation at the proximal margin

## Discussion

The present systematic review included six observational cohorts [19–24], totalizing 1062 patients with diverticular disease. Recurrence occurred in 62 cases and needed conservative (43 cases) or surgical (14 cases) treatment. Three variables were significantly associated with postoperative recurrence of diverticulitis, one for each pre-, peri- or postoperative category. From the eight preoperative variables, a lower age [20] was associated with recurrence. From the eight perioperative factors, the anastomotic level was significant on regression analysis. Three studies [20, 21, 24] integrated four postoperative variables, of which persistent postoperative pain [20] was associated with recurrence on Cox regression model.

Our review had several limitations. Firstly, regression analysis was not undertaken by all the included studies. Secondly, risk of bias was high due to the design of the included studies (one prospective [22], four retrospectives [19–21, 24], and one mixed [23] observational cohorts). Thirdly, the study populations were small, and only one study [24] was multicentric. Fourthly, data were heterogeneous across studies (i.e. severity staging of the diverticulitis, indication fur surgery, operative technique, and definition of complications). Fifthly, the search strategy may have not retrieved all relevant studies.

Importantly, diverticulosis in limited to the descending colon and sigmoid in > 90% of cases [1] and sigmoidectomy seemed a good option for the treatment of diverticulitis [5]. However, it might not be a definitive cure for all patients, as showed by a cumulative time-related incidence of postoperative recurrence at 15 years ranging between 6.3 and 16% [20, 21]. Risk factors for recurrence should be identified, to avoid increased costs and morbidity. A systematic review by Hupfeld et al. [27] identified three factors with high likelihood to increase the risk of diverticulitis recurrence after non-surgical management: young age, diverticulitis complicated by an abscess formation, and recurrent diverticulitis. Compared with the latter review [27], we presently included two studies [20, 21] which assessed the relationship between age and postoperative recurrence. While one study [21] failed to find an association, another study [20] showed decreased recurrence in older patients (HR = 0.96, 95 % CI 0.93–0.99, *p* = 0.02). This might be explained by the decreased life expectancy while reappearance of diverticulitis could occur.

Herein, we presented the first systematic review of variables associated with postoperative recurrence. Identification of these factors could help optimization of the treatment strategy. From six identified variables, only the anastomotic level is modifiable. Based on a low level of evidence, the EAES and SAGES recommended colorectal anastomosis to decrease the risk of postoperative recurrence. This statement is supported by the study by Thaler et al. [24] reporting increased recurrences with colosigmoid anastomosis *versus* colorectal anastomosis. However, Andeweg et al. [20] showed increased recurrences with colorectal anastomosis *versus* colostomy, and Regenet *et al.* [23] found no association between the anastomotic level and postoperative recurrence. Because the results are conflictual, we could not favor an anastomotic level over another. Moreover, five additional non-modifiable risk factors were identified. Because elective sigmoidectomy is associated with postoperative complication rate of 22.5% and 30-day mortality rate of 0.5% [28], benefices should be weight against the risks. This balance should consider postoperative recurrence and associated risk factors, together with the patient preferences and global condition.

Future researches are needed to identify risk factors for postoperative recurrence. Our review reported conflicting results, and significant association between variable and recurrence were reported by isolated study. Moreover, future trials should include larger prospective cohorts.

## Conclusions

To conclude, surgeons should be aware of the risk of postoperative diverticulitis recurrence, and patients should be informed. Preoperative variables associated with postoperative recurrence should be considered by clinicians for adequate patient selection and aid surgical decision-making for elective sigmoidectomy. Moreover, peri- and postoperative variables should be emphasized for optimal patient follow-up and early recognition of recurrence to avoid complication and reoperation.

## Electronic supplementary material

ESM 1 (DOC 70 kb)

## Data Availability

The authors confirm that the data supporting the findings of this study are available within the article.

## References

[1] Jacobs DO (2007). Clinical practice. Diverticulitis. N Engl J Med.

[2] Peery AF, Barrett PR, Park D, Rogers AJ, Galanko JA, Martin CF, Sandler RS (2012). A high-fiber diet does not protect against asymptomatic diverticulosis. Gastroenterology.

[3] Strate LL, Morris AM (2019). Epidemiology, pathophysiology, and treatment of diverticulitis. Gastroenterology.

[4] Wasvary H, Turfah F, Kadro O, Beauregard W (1999). Same hospitalization resection for acute diverticulitis. Am Surg.

[5] Francis NK, Sylla P, Abou-Khalil M, Arolfo S, Berler D, Curtis NJ, Dolejs SC, Garfinkle R, Gorter-Stam M, Hashimoto DA, Hassinger TE, Molenaar CJL, Pucher PH, Schuermans V, Arezzo A, Agresta F, Antoniou SA, Arulampalam T, Boutros M, Bouvy N, Campbell K, Francone T, Haggerty SP, Hedrick TL, Stefanidis D, Truitt MS, Kelly J, Ket H, Dunkin BJ, Pietrabissa A (2019). EAES and SAGES 2018 consensus conference on acute diverticulitis management: evidence-based recommendations for clinical practice. Surg Endosc.

[6] Regenbogen SE, Hardiman KM, Hendren S, Morris AM (2014). Surgery for diverticulitis in the 21st century: a systematic review. JAMA Surg.

[7] Mizrahi I, Al-Kurd A, Chapchay K, Ag-Rejuan Y, Simanovsky N, Eid A (2018). Long-term outcomes of sigmoid diverticulitis: a single-center experience. J Surg Res.

[8] Frattini J, Longo WE (2006). Diagnosis and treatment of chronic and recurrent diverticulitis. J Clin Gastroenterol.

[9] Moher D, Liberati A, Tetzlaff J, Altman DG, PRISMA Group (2009). Preferred reporting items for systematic reviews and meta-analyses: the PRISMA statement. PLoS Med.

[10] Thaler K, Weiss E, Nogueras J, Arnaud JP, Wexner S, Bergamaschi R (2003). Recurrence rates at minimum 5-year follow-up: laparoscopic versus open sigmoid resection for uncomplicated diverticulitis. Surg Laparosc Endosc Percutan Tech.

[11] Lee IK, Lee YS, Kim SJ, Gorden DL, Won DY, Kim HJ, Cho HM, Jeon HM, Kim JG, Oh ST (2010). Laparoscopic and open surgery for right colonic diverticulitis. Am Surg.

[12] Gregersen R, Andresen K, Burcharth J, Pommergaard H-C, Rosenberg J (2018). Long-term mortality and recurrence in patients treated for colonic diverticulitis with abscess formation: a nationwide register-based cohort study. Int J Color Dis.

[13] Benn PL, Wolff BG, Ilstrup DM (1986). Level of anastomosis and recurrent colonic diverticulitis. Am J Surg.

[14] Gervaz P, Mugnier-Konrad B, Morel P, Huber O, Inan I (2011). Laparoscopic versus open sigmoid resection for diverticulitis: long-term results of a prospective, randomized trial. Surg Endosc.

[15] Klarenbeek BR, Bergamaschi R, Veenhof AAFA, van der Peet DL, van den Broek WT, de Lange ESM, Bemelman WA, Heres P, Lacy AM, Cuesta MA (2011). Laparoscopic versus open sigmoid resection for diverticular disease: follow-up assessment of the randomized control Sigma trial. Surg Endosc.

[16] Letarte F, Hallet J, Drolet S, Charles Grégoire R, Bouchard A, Gagné J-P, Thibault C, Bouchard P (2013). Laparoscopic emergency surgery for diverticular disease that failed medical treatment: a valuable option? Results of a retrospective comparative cohort study. Dis Colon Rectum.

[17] Turunen P, Wikström H, Carpelan-Holmström M, Kairaluoma P, Kruuna O, Scheinin T (2010). Smoking increases the incidence of complicated diverticular disease of the sigmoid colon. Scand J Surg.

[18] Thörn M, Graf W, Stefànsson T, Påhlman L (2002). Clinical and functional results after elective colonic resection in 75 consecutive patients with diverticular disease. Am J Surg.

[19] Bergamaschi R, Arnaud JP (1998). Anastomosis level and specimen length in surgery for uncomplicated diverticulitis of the sigmoid. Surg Endosc.

[20] Andeweg C, Peters J, Bleichrodt R, van Goor H (2008). Incidence and risk factors of recurrence after surgery for pathology-proven diverticular disease. World J Surg.

[21] Choi KK, Martinolich J, Canete JJ, Valerian BT, Chismark DA, Ata A, Lee EC (2020). Elective Laparoscopic Sigmoid Colectomy for Diverticulitis-an Updated Look at Recurrence After Surgery. J Gastrointest Surg.

[22] Holmer C, Lehmann KS, Engelmann S, Gröne J, Buhr HJ, Ritz J-P (2011). Long-term outcome after conservative and surgical treatment of acute sigmoid diverticulitis. Langenbeck's Arch Surg.

[23] Regenet N, Pessaux P, Tuech J-J, Hennekinne S, Lermite E, Ridereau-Zins C, Aube C, Bergamaschi R, Jean-Pierre A (2005). Prospective evaluation of the quality of laparoscopic sigmoid resection for diverticular disease. Hepatogastroenterology..

[24] Thaler K, Baig MK, Berho M, Weiss EG, Nogueras JJ, Arnaud JP, Wexner SD, Bergamaschi R (2003). Determinants of recurrence after sigmoid resection for uncomplicated diverticulitis. Dis Colon Rectum.

[25] Hinchey EJ, Schaal PG, Richards GK (1978). Treatment of perforated diverticular disease of the colon. Adv Surg.

[26] Hansen O, Graupe F, Stock W (1998). Prognostic factors in perforating diverticulitis of the large intestine. Chir Z Alle Geb Oper Med.

[27] Hupfeld L, Burcharth J, Pommergaard H-C, Rosenberg J (2017). Risk factors for recurrence after acute colonic diverticulitis: a systematic review. Int J Color Dis.

[28] Haas JM, Singh M, Vakil N (2016). Mortality and complications following surgery for diverticulitis: Systematic review and meta-analysis. United European Gastroenterol J.

